# Focused Ultrasound-Enhanced Delivery of Intranasally Administered Anti-Programmed Cell Death-Ligand 1 Antibody to an Intracranial Murine Glioma Model

**DOI:** 10.3390/pharmaceutics13020190

**Published:** 2021-02-01

**Authors:** Dezhuang Ye, Jinyun Yuan, Yimei Yue, Joshua B. Rubin, Hong Chen

**Affiliations:** 1 Department of Mechanical Engineering and Materials Science, Washington University in St. Louis, St. Louis, MO 63130, USA; dezhuang.ye@wustl.edu; 2 Department of Biomedical Engineering, Washington University in St. Louis, St. Louis, MO 63130, USA; jinyun.yuan@wustl.edu (J.Y.); yue.y@wustl.edu (Y.Y.); 3 Department of Pediatrics, Washington University School of Medicine, St. Louis, MO 63110, USA; rubin_j@wustl.edu; 4 Department of Neuroscience, Washington University School of Medicine, St. Louis, MO 63110, USA; 5 Department of Radiation Oncology, Washington University School of Medicine, St. Louis, MO 63108, USA

**Keywords:** focused ultrasound, intranasal delivery, brain drug delivery, immune checkpoint inhibitor, blood-brain barrier, brainstem glioma

## Abstract

Immune checkpoint inhibitors have great potential for the treatment of gliomas; however, their therapeutic efficacy has been partially limited by their inability to efficiently cross the blood–brain barrier (BBB). The objective of this study was to evaluate the capability of focused-ultrasound-mediated intranasal brain drug delivery (FUSIN) in achieving the locally enhanced delivery of anti-programmed cell death-ligand 1 antibody (aPD-L1) to the brain. Both non-tumor mice and mice transcranially implanted with GL261 glioma cells at the brainstem were used in this study. aPD-L1 was labeled with a near-infrared fluorescence dye (IRDye 800CW) and administered to mice through the nasal route to the brain, followed by focused ultrasound sonication in the presence of systemically injected microbubbles. FUSIN enhanced the accumulation of aPD-L1 at the FUS-targeted brainstem by an average of 4.03- and 3.74-fold compared with intranasal (IN) administration alone in the non-tumor mice and glioma mice, respectively. Immunohistochemistry staining found that aPD-L1 was mainly located within the perivascular spaces after IN delivery, while FUSIN further enhanced the penetration depth and delivery efficiency of aPD-L1 to the brain parenchyma. The delivered aPD-L1 was found to be colocalized with the tumor cells after FUSIN delivery to the brainstem glioma. These findings suggest that FUSIN is a promising technique to enhance the delivery of immune checkpoint inhibitors to gliomas.

## 1. Introduction

Immune checkpoint inhibitor (ICI) therapy has revolutionized the field of cancer immunotherapy. Under normal physiological conditions, immune checkpoints are crucial to maintaining immune tolerance. However, in the tumor environment, tumor cells hijack these inhibitory mechanisms to avoid antitumor immune responses. ICIs are monoclonal antibodies that disrupt the engagement of immune checkpoints, which enables tumor-reactive T cells to overcome inhibitory mechanisms and mount effective antitumor immune responses [1]. PD-1 is an immune checkpoint protein expressed on the T-cell surface. PD-1 binds to the overexpressed PD-L1 receptors on the tumor cells and can silence the T cell’s function of killing the tumor cell. During ICI therapy, an anti-PD-1 antibody (aPD-1) or anti-PD-L1 antibody (aPD-L1) can bind to the respective PD-1 or PD-L1 proteins, blocking them from binding to each other and enabling the T cell to kill the tumor cell. Preclinical studies have demonstrated some efficacy against cancers by aPD-1 and/or aPD-L1, either as monotherapy or in conjunction with standard-of-care treatments [2]. However, as summarized in a recent review article [3], all reported clinical trials of ICI therapy in glioma patients failed to show superior results compared with the standard of care. Although the underlying mechanisms for the failure remain unclear, the blood–brain barrier (BBB) is considered to be one of the major obstacles for the ICI therapy of gliomas [4]. The BBB, which is responsible for maintaining a constant brain microenvironment, restricts the entry of ICIs into the brain and limits the efficacy of ICI immunotherapy.

Several strategies have been proposed to enhance the delivery of ICIs across the BBB. One strategy modified the aPD-L1 by attaching BBB targeting moieties on the antibody, which facilitated the trans-BBB delivery of systemically injected aPD-L1 by dopamine-receptor-mediated transcytosis [5]. Another study loaded aPD-1 and aCTLA-4 to a polymer-drug carrier, poly (β-L-malic acid), to enhance their permeability through the BBB by transferrin receptor (TfR)-mediated transcytosis [6]. Both approaches enhanced ICI delivery to glioma, led to enhanced immune response, and prolonged animal survival. Although promising, the chemical modifications have two main limitations: the ICIs were delivered to the whole brain instead of targeting the glioma, and systemic administration of the ICIs led to systemic toxicity [7]. Besides modifying the ICIs, an ongoing clinical trial delivers ICIs directly into the surgical resection cavity of the recurrent glioma (clinicaltrial.gov; NCT03233152), allowing direct delivery of ICIs to the tumor. However, this delivery strategy is invasive, and the penetration depth of the delivered ICIs into the surrounding brain tissue is limited [8].

Focused ultrasound (FUS)-mediated intranasal delivery (FUSIN) harvests the unique advantages of intranasal (IN) administration for direct nose-to-brain drug administration with minimized systemic exposure and of FUS in combination with microbubbles for targeted and enhanced delivery to the brain [9,10,11,12]. FUS-induced microbubble cavitation pushes and pulls on the adjacent blood vessel [13], generating the hypothesized “microbubble pumping effect”. The microbubble pumping effect is hypothesized to enhance the penetration and accumulation of IN-administered drugs. FUSIN has been utilized to deliver multiple agents, including dextrans, gold nanoparticles, and protein drugs to the FUS-targeted region with high efficiency and minimized systemic exposure [10,12,14,15]. The previous work suggests that FUSIN is a promising brain drug delivery technique that can enhance the delivery efficiency of different agents to different brain regions; however, no study has been performed to evaluate the potential of FUSIN in the delivery of ICIs to the glioma.

The objective of this study was to demonstrate the potential of FUSIN in the noninvasive and targeted delivery of aPD-L1 to an intracranial murine glioma model. A near-infrared fluorescent dye (IRDye 800CW) was conjugated to aPD-L1 (800CW-aPD-L1) for direct visualization of the spatial distribution of aPD-L1 in ex vivo brain. The IRDye 800CW has low autofluorescence background, making it an attractive option for antibody labeling. FUSIN delivery of 800CW-aPD-L1 was first evaluated in non-tumor mice, followed by studies using mice with orthotopic implantation of murine glioma cells (GL261) at the brainstem. Treatment of the brainstem glioma is extremely challenging. The brainstem connects the brain to the spinal cord and contains densely packed ascending and descending tracts and nuclei carrying information to and from the brain [16,17]. The brainstem is responsible for controlling many pivotal body functions, such as blood pressure, respiration, swallowing, motor skills, sensory activity, coordination, and walking. The critical anatomic location and functions of the brainstem hamper the application of invasive techniques for glioma treatment, such as neurosurgery. Although chemotherapy and/or radiation therapy are more often used, they are rarely curative [18]. Thus, effective delivery techniques that can noninvasively overcome the BBB are crucial for enhancing the efficacy of ICI therapy for brainstem glioma. This study showed that FUSIN noninvasively enhanced the accumulation of aPD-L1 at the brainstem in both non-tumor mice and glioma mice. Our findings suggest that FUSIN is a promising technique to enhance the delivery of ICIs to brain gliomas.

## 2. Materials and Methods

### 2.1. Near-Infrared Fluorescent Dye-Labeled aPD-L1

Monoclonal aPD-L1 was obtained from Bio X Cell (West Lebanon, NH, USA) and diluted to 2.4 mg/mL when ready to use. IRDye 800CW with NHS ester was purchased from LI-COR Biosciences (Lincoln, NE, USA). IRDye 800CW was conjugated to aPD-L1 following the manufacturer’s protocol for labeling high-molecular-weight proteins (LI-COR Biosciences). In brief, aPD-L1 was brought to pH 8.5 with 1 M potassium phosphate (pH 9) and mixed with the dye in a 3:1 dye/aPD-L1 mol/mol ratio. The antibody/dye solution was protected from light and incubated for 2 h at room temperature. The labeled aPD-L1 was purified using a desalting column (7000 MWCO, Thermo Fisher Scientific, Logan, UT, USA) pre-equilibrated with 1× PBS. The conjugated aPD-L1 was collected in the 1× PBS and kept at 4 °C until further use. The dye-to-protein ratio of the conjugate was determined according to the manufacturer’s protocol (LI-COR Biosciences) by measuring the absorbance of 800CW-aPD-L1 at 280 nm (*A*_280_) and 780 nm (*A*_780_) with a UV spectrophotometer (UV-2450 Shimadzu, Kyoto, Japan). The conjugate was diluted in a mixture of PBS and methanol (1:1), and the following formula was used for calculating the dye/protein (*D*/*P* in mol/mol) ratio,

$$\frac{D}{P}=\left[\frac{A_{780}}{\varepsilon_{Protein}}\right]÷\left[\frac{A_{280}-\left(0.03\times A_{780}\right)}{\varepsilon_{Dye}}\right]$$

where 0.03 is a correction factor for the absorbance of the IRDye 800CW at 280 nm (equal to 3.0% of its absorbance at 780 nm), while *ε_Dye_* and *ε_Protein_* are molar extinction coefficients for the dye and protein, respectively. *ε_Dye_* is 270,000 M^−1^ cm^−1^ and *ε_Protein_* is 203,000 M^−1^ cm^−1^ for typical immunoglobulin G antibodies (IgG) in a 1:1 mixture of PBS/methanol [19].

### 2.2. Confirmation of Fluorescence Labeling

Sodium dodecyl sulfate polyacrylamide gel electrophoresis (SDS-PAGE) was performed to confirm the conjugation of the aPD-L1 with IRDye 800CW. aPD-L1 and 800CW-aPD-L1 (10 μg of each) were boiled and reduced with 4× Laemmli sample buffer (Bio-Rad, Hercules, CA, USA) containing β-mercaptoethanol. Electrophoresis was performed on 4–15% Mini-PROTEAN^®^ TGX™ Precast Protein Gels (Bio-Rad, Hercules, CA, USA). The gel was imaged with the Licor Pearl small animal imaging system (LI-COR Biosciences) using the 800 nm channel, then stained with Coomassie blue overnight and imaged with a ChemiDoc™ MP Imaging System (Bio-Rad, Hercules, CA, USA) using white light.

### 2.3. 800CW-aPD-L1 Binding Assay with Flow Cytometry

To evaluate whether 800CW labeling affected the capability of aPD-L1 binding to PD-L1, GL261 cells (which had high surface PD-L1 expression [20]) were used to perform a binding assay with 800CW-aPD-L1 using flow cytometry. Briefly, GL261 cells (cell count: ~2 × 10^5^) were collected and suspended with 100 μL FACS buffer (0.5% BSA + 2 mM EDTA/PBS). Cells were stained with 1 µg aPD-L1 and 800CW-aPD-L1 at 4 °C for 30 min. Cells without aPD-L1 staining were used as control. After that, all cells were then incubated with 1 µg Alexa Fluor^®^ 647 conjugated anti-rat IgG (Cell Signaling Technology, Beverly, MA, USA) at 4 °C for 30 min. After incubation, cells were washed and resuspended with FACS buffer for measurement with flow cytometry. Data were acquired using MACSQuant (Miltenyi Biotec, Surrey, UK) and analyzed using Flowjo software (Treestar Inc., Ashland, OR, USA).

### 2.4. Animals

All animal procedures were reviewed and approved by the Institutional Animal Care and Use Committee in accordance with the National Institutes of Health guidelines for animal research (approval no. 20180186; date of approval: 12 August 2019). Cr. NIH Swiss mice (6–8 weeks, ~25 g body weight, female) were purchased from Charles River Laboratory (Wilmington, MA, USA). The animals were housed in a room maintained at 22 °C and 55% relative humidity, with a 12-h/12-h light/dark cycle and access to standard laboratory chow and water. Both mice without and with glioma implantation were used in this study.

### 2.5. Intracranial Glioma Model

For the glioma mice, the mouse glioma cell line GL261 with the expression of the enhanced green fluorescent protein (GL261-eGFP) was obtained from Dr. Dinesh Thotala (Washington University School of Medicine, St. Louis, MO, USA) and cultured in Dulbecco’s Modified Eagle Medium (DMEM) with Nutrient Mixture F-12 1:1, 10% fetal bovine serum, and 1% sodium pyruvate (Life Technologies, Carlsbad, CA, USA) in a 5% CO_2_ incubator at 37 °C. Mice were anesthetized and their heads fixed on a stereotactic frame. A paramedian incision was made on the scalp, and a 1-mm burr hole was drilled 0.8 mm posterior and 1.0 mm lateral to the lambda. GL261-eGFP cells (volume: 2 µL; cell count: 5 × 10^4^) were injected through the burr hole using a syringe. The burr hole was sealed with bone wax, and the skin incision was glued together with tissue glue. Post-surgery analgesia was provided by subcutaneous injection of buprenorphine (0.03 mg/kg in saline twice daily) for 3 days. The FUSIN (*n* = 5) and IN (*n* = 5) delivery of 800CW-aPD-L1 to the glioma mice was conducted on day 14 post-implantation.

### 2.6. FUSIN Treatment

Both non-tumor mice and glioma mice were used for FUSIN delivery following a similar procedure, as reported before [14]. Briefly, mice were placed supine under anesthesia. Drops (3 µL each) of 800CW-aPD-L1 were administered to the mouse nose, alternating between the left and right nostrils every 2 min. A total of 8 drops (24 µL) were administered to each mouse. The concentration of the 800CW-aPD-L1 solution was 0.8 mg/mL for non-tumor mice and increased to 2.4 mg/mL for the glioma mice. The lower concentration of the 800CW-aPD-L1 applied on the non-tumor mice was used to prove the feasibility of 800CW-aPD-L1 delivery by FUSIN. The concentration was increased when delivered to glioma mice to better match the dose used in clinical studies [21,22,23]. After IN administration, the mouse head was then stabilized on a stereotaxic frame, and hairs on the head were removed for the FUS treatment. An ultrasound image-guided FUS system (VIFU 2000; Alpinion US Inc., Bothell, WA, USA) consisting of an ultrasound imaging probe (L8-17, Alpinion, Seoul, Korea) and an FUS transducer (center frequency 1.5 MHz) was used to target the FUS at the brainstem under the assistance of a metal grid [24]. Size-isolated microbubbles (median diameter: 4–5 µm; concentration: ~8 × 10^8^ #/mL; injection volume: 30 μL) manufactured in-house [25] were injected through the tail vein, immediately followed by FUS sonication. FUS sonication was performed at 0.5 h after IN administration using the following parameters: pressure = 0.43 MPa, pulse length = 6.7 ms, pulse repetition frequency = 5 Hz, and duration = 1 min. Four points located at the corners of a square with a side length of 0.6 mm were treated to enlarge the treatment volume [14]. Mice were transcardially perfused at 1 h post-IN delivery, which is ~0.5 h after FUS sonication. Mice delivered by IN only were used as the control and sacrificed at 1 h post-IN delivery.

### 2.7. Ex Vivo Fluorescence Imaging and Quantification

After perfusion, the brains were excised and sliced into 2-mm coronal sections using a brain matrix (RBM-2000C; ASI Instruments, Inc., Warren, MI, USA) and examined by the Licor Pearl small animal imaging system, with acquisition using the 800 nm channel for 800CW-aPD-L1. The exposure time for fluorescence imaging was kept the same (30 s) for all groups. The fluorescence intensity of the brain slices was quantified using Licor’s Image Studio Lite software.

### 2.8. ELISA Quantification of 800CW-aPD-L1 Delivery Efficiency

ELISA quantification of the 800CW-aPD-L1 delivery was performed for the non-tumor mice treated by IN only (*n* = 5) or FUSIN (*n* = 5) to verify the fluorescence quantification by the Licor Pearl system. After imaging by the Licor Pearl system, the mouse brainstem tissues were collected, weighed, and homogenized by adding 1× RIPA buffer at 10× tissue weight. The homogenized tissues were then centrifuged at 5000× *g* for 10 min at 4 °C and the supernatant was collected. The protein concentration of the supernatant was measured with BCA assay and diluted to 1 mg/mL using dilution buffer (PBS+/0.5% BSA+0.05% Tween-20). The ELISA standard was made using the aPD-L1 stock to generate a serial dilution in the dilution buffer. The ELISA plates (Thermo Fisher Scientific, Wilmington, DE, USA) were coated with recombinant mouse PD-L1 protein (Cat. No. PD1-M5251; ACROBiosystems, Beijing, China) at 4 °C overnight. The plates were then washed with 300 µL washing buffer (PBS+0.05% Tween-20) three times and blocked with blocking buffer (PBS+2% BSA+0.05% Tween-20) for 1.5 h at room temperature. The tissue supernatant and the aPD-L1 standards were added to the pre-coated plate and incubated overnight at 4 °C, and the polyclonal horseradish peroxidase (HRP)-conjugated rabbit anti-rat antibodies (Jackson ImmunoResearch, West Grove, PA, USA) were then added and incubated for 1.5 h at room temperature. After the incubation, the plates were washed and added with 90 µL TBM substrates for 10 min, followed by 50 µL stop buffer. Data was acquired with SpectraMax^®^ i3 Platform (Molecular Devices LLC, San Jose, CA, USA). The concentration of 800CW-aPD-L1 in brainstem tissue samples was calculated in reference to the 800CW-aPD-L1 standard curve.

### 2.9. Immunofluorescence Staining

In order to further study the microscopic level distributions of 800CW-aPD-L1 after FUSIN, the mouse brainstem after FUSIN was harvested and stained for the aquaporin 4 (AQP4), the blood vessel, and the aPD-L1. AQP4 is the most prevalent aquaporin channel, specifically located at the perimicrovessel astrocytic endfeet and aligned with the perivascular spaces. The brainstems of tumor mice were also harvested and stained for aPD-L1 to image the spatial distribution of the delivered aPD-L1. All the brain samples were fixed in 4% paraformaldehyde (PFA) overnight and equilibrated in 30% sucrose for cryosectioning. The fixed brainstems were sectioned into 20 µm slices using a Leica CM3050 S cryostat (Leica Biosystems). The slices were preprocessed in 0.3% *v*/*v* Triton X-100 and 3% *v*/*v* blocking serum solution in PBS for 1 h in the dark at room temperature to increase the permeability and block the background, then washed using PBS. For the non-tumor mice, the slices were incubated in an anti-aquaporin4 antibody (anti-AQP4) (Alomome Lab, Cat: AQP-004, 1:200) solution overnight at 4 °C, washed with PBS, and then incubated in Alexa Fluor 594 AffiniPure donkey anti-rat IgG (H+L) (Jackson immunoResearch, Cat: 712-585-153, 1:400) overnight at 4 °C for staining aPD-L1. After that, the slices were added with Alexa Fluor 488 donkey anti-rabbit IgG (Jackson immunoResearch, Cat: 711-545-152, 1:400) and DyLight 649-labeled tomato lectin (Vector Laboratories, Cat: DL-1178-1, 1:1000) and incubated in the dark for 2 h at room temperature. Finally, the slices were mounted with VECTASHIELD antifade mounting media (Vector Laboratories). For the tumor mice, the slices were only stained with Alexa Fluor 594 AffiniPure donkey anti-rat IgG (H+L), following the same procedure mentioned above.

### 2.10. Statistical Analysis

Statistical analysis was performed using GraphPad Prism (Version 8.3, La Jolla, CA, USA). Differences between the two groups (IN vs. FUSIN) were determined using an unpaired two-tailed Student’s *t*-test. A *p*-value < 0.05 was used to determine statistical significance.

## 3. Results

### 3.1. Characterization of 800CW-aPD-L1

**Figure 1:**
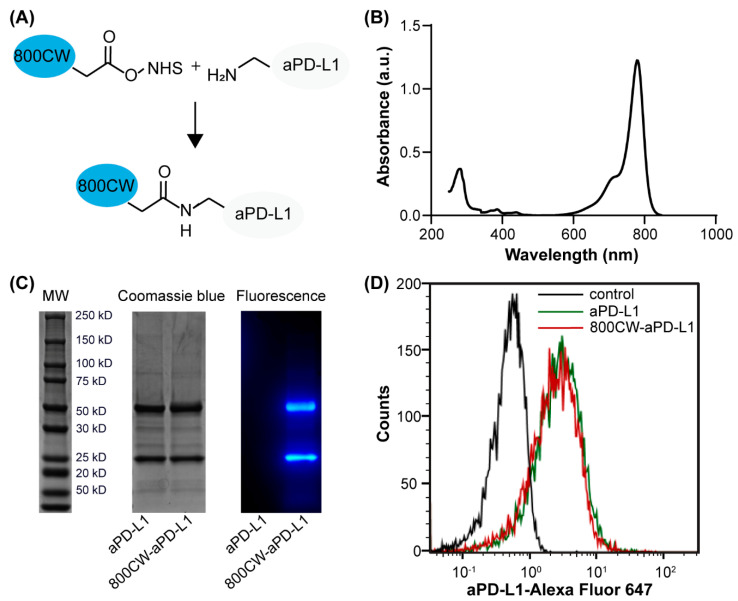
Analysis of 800CW-aPD-L1 conjugates. (**A**) Schematic illustration of 800CW conjugated to aPD-L1 through NHS ester reaction. (**B**) Absorbance spectrum of 800CW-aPD-L1 measured by the UV spectrophotometer. (**C**) SDS-PAGE of reduced aPD-L1 and 800CW-aPD-L1 by both Coomassie blue staining and fluorescence imaging. (**D**) Flow cytometry analysis of binding affinity of aPD-L1 and 800CW-aPD-L1 to GL261 cells with high surface PD-L1 expression (black: unstained control, red: 800CW-aPD-L1, green: aPD-L1).

The 800CW-aPD-L1 conjugation (Figure 1A) had an average of 2.7 molecules of 800CW dye per single molecule of aPD-L1, as determined by the UV spectrophotometer (Figure 1B). Furthermore, the reduced SDS-PAGE conditions produce heavy chains (∼50 kDa) and light chains (∼25 kDa) of the aPD-L1. The increase in the molecular weight displayed by SDS-PAGE stained by Coomassie blue suggested the 800CW were bound to both the light chain and the heavy chain of aPD-L1. The flow cytometry analysis observed similar enhanced fluorescence intensity of cells stained with both 800CW-aPD-L1 and aPD-L1, meaning that the 800CW-aPD-L1 and aPD-L1 were bound to the GL261 cells at similar binding efficiency (Figure 1D), indicating the 800CW fluorescence labeling would not affect the capability of aPD-L1 binding to the tumor cells.

### 3.2. FUSIN Delivery of 800CW-aPD-L1 to Non-Tumor Mice

**Figure 2:**
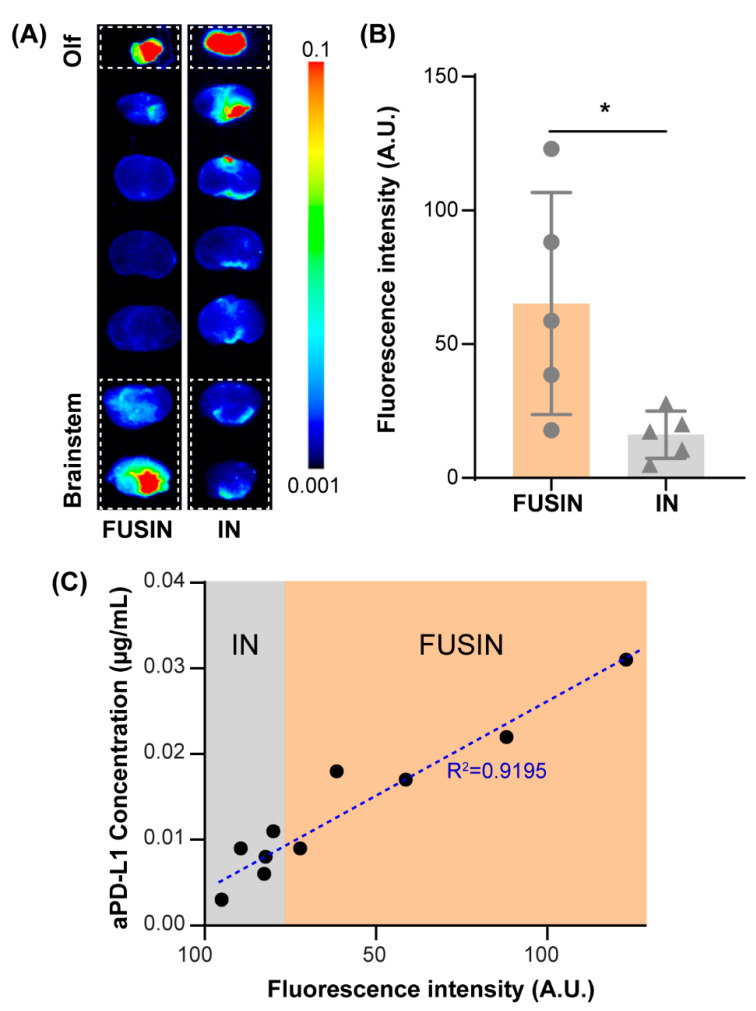
Focused-ultrasound-mediated intranasal (FUSIN) delivery of 800CW-aPD-L1 to the brainstem. (**A**) Fluorescence images of representative ex vivo mouse brain slices. The olfactory bulb (Olf) was in the top slice. The targeted location of the FUS was located at the right side of the brainstem. The dashed box highlights the brain slices containing the brainstem. (**B**) Fluorescence quantification of the 800CW-aPD-L1 delivery efficiency to the brainstem. (**C**) The relationship between the fluorescence intensity measured by ex vivo fluorescence imaging and the aPD-L1 concentrations measured by ELISA (*: *p* < 0.05).

Representative fluorescence images of the mouse brain slices from the non-tumor mice and the corresponding quantification of the delivery efficiencies are presented in Figure 2. The top slice contained the olfactory bulb. Enhanced fluorescence intensity was observed in the top slices for both FUSIN and IN groups, confirming successful IN administration of the 800CW-aPD-L1. The bottom two slices contained the brainstem. The fluorescence intensity of the bottom two slices from the FUSIN group was significantly higher than those from the IN group. Based on the fluorescence quantification, the delivery efficiency of FUSIN to the brainstem was on average 4.03-fold higher than that of IN only (*p* < 0.05). Moreover, the ELISA quantification of the same brainstem slices found a strong linear correlation (R^2^ = 0.9195) between the concentration of the 800CW-aPD-L1 and the fluorescence intensity (Figure 2C), indicating that the fluorescence intensity can be used as a surrogate for quantifying the delivery efficiency of the 800CW-aPD-L1.

**Figure 3:**
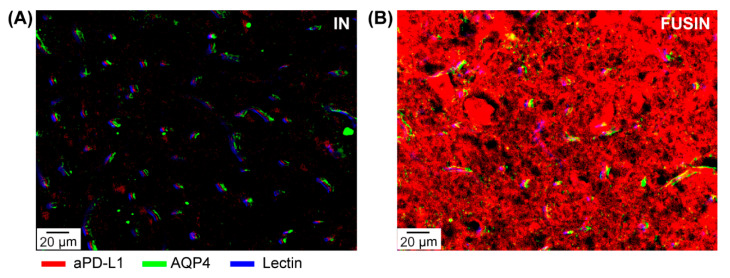
Fluorescence images of the brainstem obtained from non-tumor mice after intranasal (IN) (**A**) and FUSIN (**B**) delivery. After IN delivery, immunofluorescence staining shows the aPD-L1 distributed along with the perivascular spaces, defined by the space between the AQP4-stained astrocyte and the lectin-stained blood vessel. FUSIN enhanced the accumulation of aPD-L1, and the delivered aPD-L1 penetrated deep into the brain parenchyma.

Figure 3 shows the representative images at the microscopic level of mice brain sections after FUSIN delivery. After IN delivery, the aPD-L1 is observed between the blood vessel and the AQP4, indicating that the IN-delivered aPD-L1 were traveling along with the perivascular spaces. After FUSIN delivery, enhanced accumulation of aPD-L1 was observed, and the aPD-L1 was not only located within the perivascular spaces, but also deeply penetrated into the brain parenchyma.

### 3.3. FUSIN Delivery to Mice with Brainstem Gliomas

**Figure 4:**
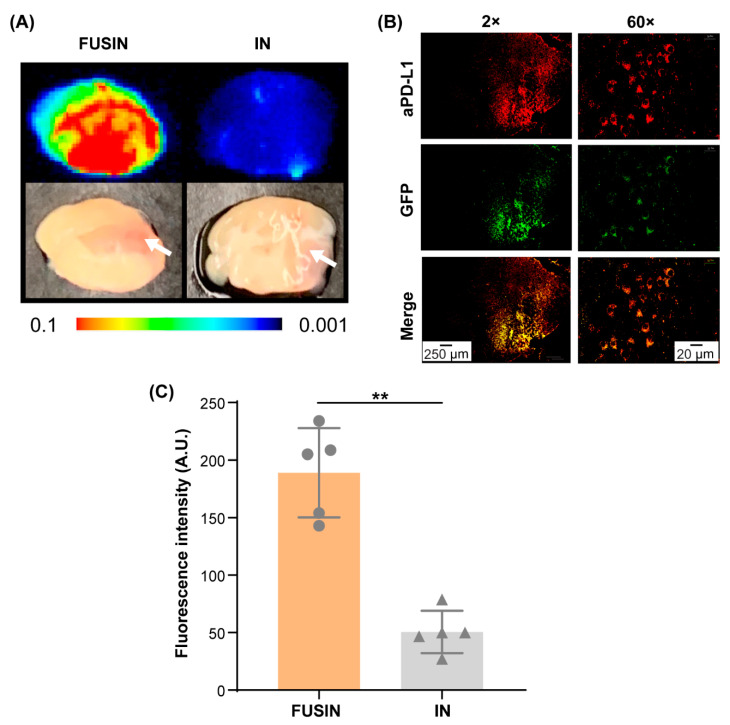
FUSIN delivery of 800CW-aPD-L1 to the brainstem glioma. (**A**) Fluorescence images of representative ex vivo mouse brainstem slices. The white arrow indicates the tumor location. (**B**) Spatial distribution of FUSIN-delivered 800CW-aPD-L1. Left panel: aPD-L1 distribution in a coronal section of the brainstem after FUSIN delivery imaged at 2×. Right panel: higher magnification view (60×) of the tumor showing the colocalization of aPD-L1 with the tumor cells. (**C**) Fluorescence quantification of the 800CW-aPD-L1 delivery efficiency to the brainstem glioma (** *p* < 0.01).

Figure 4A shows representative fluorescence images and the corresponding photographs of the brain slices of mice with brainstem gliomas. The brainstem, where the glioma was implanted, showed enhanced fluorescence signal by FUSIN compared with IN only. The location of the glioma is pointed out by the arrow. At lower magnification (2×), the aPD-L1 was observed in the FUS-targeted right side of the brainstem and locally accumulated within the glioma growing on the right side of the brainstem. At higher magnification (60×), the aPD-L1 was found to be colocalized with the tumor cells, suggesting that the delivered aPD-L1 was bound to the tumor cells (Figure 4B). The quantification of the delivery efficiency is presented in Figure 4C. The delivery efficiency of FUSIN was found to be on average 3.74-fold higher than that of IN only to the brainstem (*p* < 0.01) (Figure 4C).

## 4. Discussion

The efficient delivery of ICIs is critical for ICI therapy of the glioma. Although the glioma immune microenvironment is very complex, it has been demonstrated that the treatment efficacy of mice with gliomas could be significantly increased by enhancing the delivery of ICIs across the BBB [5,6]. The efficient delivery of ICIs to the glioma is expected to directly disrupt the PD-1/PD-L1 or CTLA-4/B7-1 complex formation in the tumor, resulting in the activation of brain local immune response and leading to the improved survival of glioma mice [26]. A previous study chemically modified the aPD-L1 for receptor-mediated transcytosis delivery across the BBB [5]. That study achieved a 2-fold increase of aPD-L1 delivery compared to aPD-L1 without modification in the GL261 mouse model after intravenous injection, and significantly improved mouse survival. In the current study, aPD-L1 was labeled by a near-infrared fluorescent dye. The low autofluorescence background of the dye and the stable labeling provide unique strengths for evaluating the delivery outcome of aPD-L1 by FUSIN. Using the 800CW-aPD-L1, we found that the delivery efficiency of FUSIN was on average 4.03-fold higher in non-tumor mice and 3.74-fold higher in glioma mice when compared to IN delivery only. This is the first study that applied FUSIN in a mouse model of glioma. The finding that the enhancement ratios were comparable for non-tumor mice and tumor mice suggests that the enhancement effect of FUSIN is not affected by the tumor microenvironment. Future study needs to examine the corresponding immune response and treatment efficacy to demonstrate the therapeutic benefits of the FUSIN delivery of aPD-L1.

The mechanism of FUSIN delivery is still under investigation. The olfactory nerve and trigeminal nerve ending in the nose cavity provide a route for direct medication delivery into the brain in nose-to-brain delivery. Bulk transport along the channels surrounding the olfactory and trigeminal nerves is the most likely mechanism for nose-to-brain transport, based on the rapid speed of transport from the nose to the brain [27]. Once inside the brain entry points (i.e., olfactory bulb and brainstem), the IN-administered agents are distributed in the whole brain, along with the cerebral perivascular spaces, and propelled through the perivascular spaces by heartbeat-driven pulsations of the blood vessel walls, which is called the perivascular pump effect [28]. Previous IN delivery of IgG was observed in the perivascular spaces, supporting that perivascular spaces are the potential transporting pathways for the brain distribution of intranasally administered antibodies [26,27]. This study showed similar results in the brainstem after IN administration of aPD-L1 without FUS treatment, confirming that the IN-delivered aPD-L1 distributed along the perivascular spaces (Figure 3A). Meanwhile, on the FUS-treated brainstem, the accumulation and penetration of the aPD-L1 were boosted (Figure 3B). Our previous work on ultra-high-speed photomicrography of microbubble dynamics in ex vivo microvessels observed that microbubble oscillations push and pull on the blood vessel, leading to expansion and contraction of the vessel and surrounding tissues [13]. Based on the similarity of this phenomenon with the perivascular pump effect, we hypothesized that the “microbubble pump effect” may be the potential mechanism for FUSIN. The microbubble pump could increase the bulk flow in the perivascular spaces and even the surrounding interstitial fluid, enhancing convective transport. Meanwhile, at the FUS-targeted region, as IN-delivered aPD-L1 transports away from the perivascular spaces to the brain parenchyma, the concentration of aPD-L1 in the perivascular spaces may decrease. Thus, the aPD-L1 in the perivascular spaces outside the FUS-targeted region may flow into the targeted region driven by a concentration gradient, leading to enhanced local accumulation of a-PD-L1 at the FUS-targeted region. The mechanism of FUSIN warrants further investigation.

## 5. Conclusions

In this study, aPD-L1 was fluorescently labeled by IRDye 800CW and used to evaluate the potential of FUSIN in ICI delivery to both non-tumor mice and glioma mice. The labeling did not alter the binding affinity of aPD-L1 with PD-L1 protein and enabled the quantification of the spatial distribution of aPD-L1 delivery by fluorescence imaging. FUSIN achieved significantly enhanced delivery of aPD-L1 to the brainstem of non-tumor mice and brainstem glioma in tumor mice. The delivered aPD-L1 penetrated deep into the brain parenchyma at the FUSIN-targeted brainstem with high local accumulation. The delivered aPD-L1 was also found to bind to the tumor cells when delivered to the brainstem glioma. These findings suggest that FUSIN is a promising technique to facilitate the delivery of immune checkpoint inhibitors to the brain.

## Data Availability

The data presented in this study are available in the paper.
